# Best Papers of 2010

**DOI:** 10.1120/jacmp.v12i3.3680

**Published:** 2011-08-14

For the past several years, the JACMP has issued awards to several manuscripts deemed by the Board of Editors to be worthy of the designation “Best Papers.” These awards are selected by a committee of the Board of Editors and are supported by grants from some of our corporate sponsors. By listing the Best Papers of 2010, I would like to honor the award winners for their contributions to the clinical practice of medical physics, and thank our corporate sponsors for their financial support.

The Elekta Award of Excellence for an Outstanding Radiation Oncology Article was awarded to Vladimir Feygelman, Geoffrey G Zhang, and Craig W Stevens for their article entitled “Initial dosimetric evaluation of SmartArc ‐ a novel VMAT treatment planning module implemented in a multi‐vendor delivery chain,” JACMP 11(3), 135–153.

The RIT Award of Excellence for the Best Imaging Article was awarded to Luciant Wolfsberger, Matthew Wagar, Paige Nitsch, Mandar Bhagwat, and Piotr Zygmanski for their article entitled “Angular dose dependence of MatriXX and its calibration,” JACMP 11(1), 241–251.

The Sun Nuclear Award of Excellence for an Outstanding Radiation Oncology Physics Article was awarded to Ahmed Abdel Rahman Eldib, Mohamed I. Elgohary, Jiajin Fan, Lihui Jin, Jinsheng Li, Charlie Ma, and Nader A. Elsherbini for their article entitled “Dosimetric characteristics of an electron multileaf collimator for modulated electron radiation therapy,” JACMP 11(2), 5–22.

The Unfors Award of Excellence for the Best Radiation Measurements Article was awarded to Thomas Frederick Boltz, William Pavlicek, Robert Paden, Markus Renno, Angela Jensen, and Metin Akay for their article entitled “An anthropomorphic beating heart phantom for cardiac X‐ray CT imaging evaluation,” JACMP 11(1), 191–199.

The Varian – Editor‐in‐Chief Award of Excellence for an Outstanding General Medical Physics Article was awarded to Salahuddin Ahmad, Daniel Johnson, Jessica R. Hiatt, D. Timothy Still, Eli E. Furhang, David Marsden, Frank Kearly, Damian A. Bernard, and Randall W. Holt for their article entitled “Comparison of tumor and normal tissue dose for accelerated partial breast irradiation using an electronic brachytherapy eBx source and an Iridium‐192 source,” JACMP 11(4), 155–161.

Congratulations to all the award winners, and thanks very much to all the award sponsors.

George Starkschall, PhD

Editor‐in‐Chief

August 15, 2011

